# Cardiovascular Activity of Ginkgo biloba—An Insight from Healthy Subjects

**DOI:** 10.3390/biology12010015

**Published:** 2022-12-21

**Authors:** Henrique Silva, Filipe Gazalho Martins

**Affiliations:** 1 Research Institute for Medicines (iMed.ULisboa), Faculdade de Farmácia, Universidade de Lisboa, Av. Prof. Gama Pinto, 1649-003 Lisbon, Portugal; 2 Department of Pharmacy, Pharmacology and Health Technologies, Faculdade de Farmácia, Universidade de Lisboa, Av. Prof. Gama Pinto, 1649-003 Lisbon, Portugal; 3 Biophysics and Biomedical Engineering Institute (IBEB), Faculdade de Ciências, Universidade de Lisboa, Campo Grande, 1749-016 Lisbon, Portugal

**Keywords:** Ginkgo biloba, vasodilation, blood pressure, organ perfusion, review

## Abstract

**Simple Summary:**

Ginkgo biloba is the oldest living tree species in the world and its extracts are among the most used herbal preparations in the prevention and treatment of cardiovascular diseases. Most clinical data on the efficacy of Ginkgo biloba is from clinical studies, with few results from healthy subjects. This paper provides a comprehensive review of the mechanisms underlying the known beneficial cardiovascular activities of Ginkgo biloba and its main compounds. This plant displays myocardial suppressant and vasorelaxant activities ex vivo. It improves perfusion in different vascular beds without significantly affecting blood pressure and heart rate. In addition, it displays an acceptable safety profile, with most reported adverse reactions constituting rare occurrences. Collectively, Ginkgo biloba impacts cardiovascular physiology, improving hemodynamics and organ perfusion. In the future, better controlled clinical studies should be performed in order to identify the target populations who could benefit the most with pharmacotherapeutic interventions involving this plant.

**Abstract:**

Ginkgo biloba is the oldest living tree species in the world. Despite less than encouraging clinical results, extracts from its leaves are among the most used herbal preparations in the prevention and treatment of cardiovascular diseases. Most data on the efficacy of Ginkgo biloba on cardiovascular disease is from clinical studies, with few results from healthy subjects. This paper aims to provide a comprehensive review of the mechanisms underlying the known beneficial cardiovascular activities of Ginkgo biloba. It displays myocardial suppressant and vasorelaxant activities ex vivo, potentiating endothelial-dependent and -independent pathways. It improves perfusion in different vascular beds, namely ocular, cochlear, cutaneous, cerebral, and coronary. Although scarce, evidence suggests that Ginkgo biloba displays a heterogeneous effect on tissue perfusion which is dependent on the individual elimination pathways. It displays an acceptable safety profile, with most reported adverse reactions constituting rare occurrences. Collectively, Ginkgo biloba positively impacts cardiovascular physiology, improving hemodynamics and organ perfusion. In the future, better controlled clinical studies should be performed in order to identify the target populations who may benefit the most from pharmacotherapeutic interventions involving Ginkgo biloba.

## 1. Introduction

Cardiovascular disease is the number one cause of death worldwide [1,2,3], and is associated with significant morbidity, disability, and decreased quality of life [2,3]. Despite the wide array of drugs available to prevent and treat cardiovascular disease, there is an increasing need to discover molecules that can be directed to novel biological targets, with improved safety and efficacy. Plant-based medicines still play an important part in the management of several types of cardiovascular diseases [4]. A large proportion of the world’s population lacks access to chemically synthesized medicines and is largely dependent on plant-based medicines/treatments [4,5]. Additionally, plant-based medicines are perceived to possess fewer adverse reactions than standard medicines, which justifies the interest in them [6,7].

**Figure 1:**
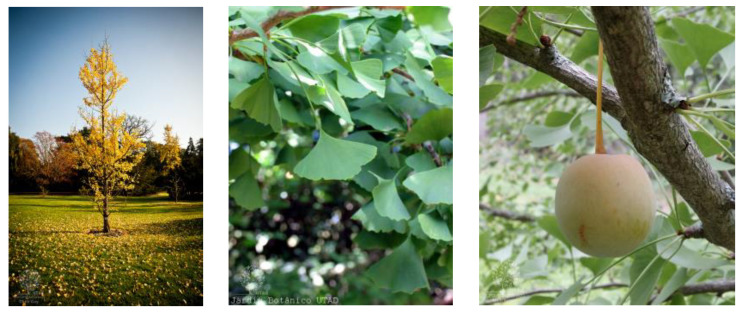
Ginkgo biloba tree: general appearance (autumn foliage; **left**); leaves (summer foliage; **center**); seeds (**right**). Photographs by Chris Guy (**left**) and Arb O’Retum (**right**) from Jardim Botânico UTAD, Flora Digital de Portugal (https://jb.utad.pt, accessed on 21 October 2022).

Ginkgo biloba, the only living species of the Ginkgophyta division, is one of the most used medicinal plants for the treatment of various diseases worldwide [8]. The name “Ginkgo” derives from an erroneous transcription of the Japanese name *Ginkyo*, meaning silver fruit [9]. The name “biloba”, refers to the bilobed shape of the leaves (Figure 1). Its common English name is maidenhair fern, due to the resemblance of the shape and veins of its leaves with those of the leaves of that plant. The Ginkgo biloba tree displays very large dimensions, reaching 20–40 m in height and 1–4 m in stem diameter. This plant is dioecious, with sexual maturity taking about 25 years. The ratio between male and female species is approximately 1:1, with occasional reports of monoecious plants. [8,10]. Due to its resistance mechanisms against pollution, insects, and bacterial and viral infections, Ginkgo biloba is an exceptionally long-lived tree, living more than a thousand years [11]. As the oldest living tree species in the world, this “living fossil” originates from the Asian continent, more specifically, eastern China (in Yangtze River Valley), Democratic People’s Republic of Korea, the Republic of Korea, and Japan [4,8,12]. Currently, it is widely cultivated in Asia, Europe, North America, New Zealand, and Argentina [13].

For centuries, Ginkgo biloba has been used in traditional Chinese medicine to treat various medical conditions [14]. It is thought to have been introduced in Europe in the 17th century through the German physician, botanist, and researcher Engelbert Kaempfer, who was the first European to describe and catalog it. Later, in 1771, the Swedish taxonomist Carl Nilsson Linnæus named the plant Ginkgo biloba [12]. In 1965, the German company Dr. Willmar Schwabe introduced the first Ginkgo biloba leaf extract. Subsequently, a standardized and concentrated purified extract was developed in collaboration with the French industry Beaufour-IPSEN. This extract is currently marketed in more than 70 countries and is widely used as a therapeutic strategy in Western medical practice [15,16]. Ginkgo biloba is among the most popular of all dietary supplements in Europe and in the USA, with total worldwide sales of Ginkgo products reaching $1.26 billion in 2012 [17].

In the last decades, Ginkgo biloba has been used clinically for the prevention and treatment of different cardiovascular diseases, including hypertension, cerebrovascular disease, peripheral arterial disease, peripheral venous disease, Raynaud’s phenomenon, and erectile dysfunction. In addition, this plant has also been used in diseases with a probable underlying vascular dysfunction component, such as cognitive decline, dementia, and tinnitus. Even though some clinical trials show positive results, the majority of systematic reviews and meta-analyses have shown less encouraging evidence. For hypertension, a recent systematic review has shown that there is no convincing evidence to support the efficacy of extracts of Ginkgo biloba (EGb) as an anti-hypertensive, due to the flawed study design and poor methodological quality of most randomized clinical trials assessed [18]. For tinnitus patients without cardiovascular disease, a recent systematic review and a meta-analysis have both reported that Ginkgo biloba does not show higher efficacy than placebo [19,20], with an exception in elderly patients with dementia [21]. For peripheral arterial disease, a systematic review and a meta-analysis have both reported that Ginkgo biloba shows higher efficacy than placebo for treating intermittent claudication [22,23]. For Raynaud’s disease, different results have been reported in double-blind placebo-controlled clinical trials. One trial reported that EGb could be effective in reducing the frequency of vascular attacks [24], whereas another reported that EGb did not reduce the duration and severity of attacks when compared with the placebo [25]. In addition, another clinical study showed that Ginkgo biloba was inferior to nifedipine in the treatment of primary Raynaud’s disease [26]. A meta-analysis that reviewed the different therapeutic approaches for Raynaud’s disease reported inconclusive evidence, due to the lack of robustness in clinical trials [27]. Regarding dementia, evidence is more encouraging. Systematic review and meta-analyses have shown that Ginkgo biloba is able to improve, stabilize or slow the progression of cognitive decline [28,29], even though more robust trials are needed [30]. For other diseases, no systematic reviews or meta-analyses have been published, with only clinical trials being available. For example, for erectile dysfunction secondary to antidepressant use, Ginkgo biloba has not shown higher efficacy than placebo [31,32].

Ginkgo biloba is popular for its ability to improve cognition, specifically attention and memory [33,34,35,36], even though convincing evidence on its efficacy is still lacking for some age groups [37,38]. Young students are known to use cognitive enhancers for improving academic performance [39,40], including Ginkgo biloba. In addition, Ginkgo biloba can be found in energy drinks, commonly consumed by teenagers and young adults, sometimes with potential health risks [41]. Similarly, Ginkgo biloba is also consumed by the elderly for preventing or delaying the onset of dementia. Considering the particular risk of polypharmacy and of herbal-drug interactions in the elderly, the efficacy of Ginkgo biloba needs to be carefully assessed.

Considering the different cellular targets of Ginkgo biloba, a deeper knowledge of the mechanisms that underlie its cardiovascular activities is much needed and highly relevant. Surprisingly, recent reviews have addressed the overall health potentials of Ginkgo biloba but have not thoroughly addressed the mechanisms underlying its cardiovascular activity [42,43,44,45,46]. Furthermore, most original studies on the cardiovascular activity of this plant in humans have been performed in clinical populations. In the few papers that address these effects on healthy subjects, hemodynamic assessment is often not their main focus. Finally, several aspects affecting the efficacy of Ginkgo biloba for treating or preventing cardiovascular disease, such as patient age, race/ethnicity, and metabolism, often contribute to heterogeneous experimental and clinical results, which could undermine current knowledge of the cardiovascular potential of this plant.

This paper aims to provide a thorough and up-to-date comprehensive review of the cardiovascular activities of Ginkgo biloba in healthy subjects, specifically on the mechanisms affecting blood pressure and hemodynamics. A special focus will be given to the currently known mechanisms for vasorelaxation. The authors intend to provide a critical discussion on the aspects that might affect the efficacy of Ginkgo biloba in order to improve drug dosing and formulation and to better identify the clinical populations who could benefit the most from this plant in the future.

## 2. Characterization of the Main Extracts of Ginkgo Biloba Leaves

The first Chinese herbal formulas considered the inner layer of the seeds of Ginkgo biloba as the active part of the plant for its therapeutic value and it is still used in Chinese medical practice to the present day [4,13]. However, from 1509 onwards, the use of Gingko biloba seeds was eventually replaced with the use of the plant’s leaves which were determined to have increased therapeutic potential. In fact, studies carried out in the last decades have confirmed that most bioactive compounds of Ginkgo biloba are contained in the leaves, from which extracts are prepared and incorporated in solid or liquid pharmaceutical formulations [4,13]. Two types of leaf extracts can be distinguished: full extracts are generally prepared with ethanol and contain all the plant’s soluble constituents and standardized extracts, which are adjusted to a defined scale of constituents [47]. Standardized extracts are defined by the quality of the raw material of the plant and by the manufacturing process, which, together, ensure a defined composition of constituents in the extract, guaranteeing the effectiveness, safety, and quality of the product [48]. According to evidence-based medicine, Ginkgo biloba should only be used in the form of a standardized extract.

Extracts of Ginkgo biloba are prepared through a process that encompasses several steps, which may differ according to the manufacturer [4,16,47]. Initially, the leaves are harvested from the trees while they are green (i.e., richer in active compounds), which maximizes the potential of the plant [49]. Once dried, the leaves are compressed into large bales to which solvents such as water, acetone, or ethanol, are added and used to produce standardized extracts [16]. After the removal of acetone, the extracts are purified by liquid-liquid extraction, thus eliminating unwanted substances, including biflavones, ginkgolic acids (anacardic acid derivatives), among others, due to their allergenic, cytotoxic and carcinogenic potential [16,47,50]. This process also increases the concentration of substances that contribute to therapeutic activity and clinical efficacy, such as flavonol glycosides and terpene trilactones [16,47].

EGb is standardized according to flavonoid or terpene trilactones content. These are characterized by having between 22% and 27% of flavonoid heterosides, represented by flavonol glycosides (kaempferol, quercetin, and isorhamnetin), between 5% and 7% of terpene trilactones (2.8–3.4% correspond to ginkgolides A, B, and C, and 2.6–3.2% consists of bilobalide), and content of less than 5 mg/kg of ginkgolic acids [51]. These extracts also contain various organic acids, responsible for increasing the water solubility of flavonoids and terpenoids, which is crucial for their therapeutic activity [52,53]. The EGb 761^®^ (Willmar Schwabe Pharmaceuticals, Karlsruhe, Germany) and LI 1370^®^ (Lichtwer Pharma, Germany) extracts are the ones considered ideal for the production of herbal medicines [16,48,53], with the former being the most used [4,49]. The EGb 761^®^ extract is constituted of 5–10% organic acids, 24% flavonol glycosides, and 6% terpene trilactones (3.1% correspond to ginkgolides A, B, C, and J, and 2.9% consists of bilobalide), with ginkgolide J being present in low concentrations [54]. Proanthocyanidins are also present in a significant concentration in the extract, being largely composed of dimers and oligomers of delphinoidine and cyanidine [52,55]. The main classes of the chemical compounds present in EGb 761^®^ extract, as well as their concentrations, are presented in Table 1. LI 1370^®^ (Lichtwer Pharma GmbH, Berlin, Germany) is standardized in comparable percentages (25% flavonol glycosides and 6% terpene trilactones), with no EGb containing biflavones [48,53].

## 3. Characterization of Bioactive Compounds (Ginkgolides, Bilobalide) in Ginkgo Biloba Leaves

Ginkgo biloba leaves have a wide variety of biologically active compounds, with flavonol glycosides (kaempferol, quercetin, and isorhamnetin), diterpenes, and sesquiterpenes being the main components responsible for the pharmacological activity associated with this plant [49,51,53]. Ginkgolides A, B, C, and J, and bilobalide are the predominant terpenoids in the leaves of this plant which are found exclusively in this species [13,50,56,57].

**Figure 2:**
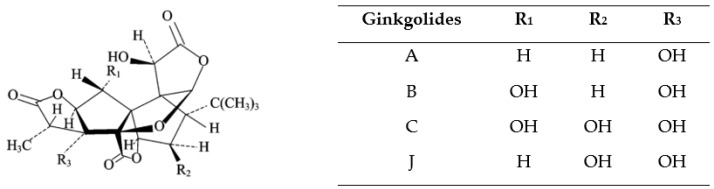
Chemical structure of ginkgolides A, B, C, and J.

Ginkgolides are diterpenes consisting of six, five-membered rings, containing a spiro[4,4]nonane system, three lactones, and a tetrahydrofuran, incorporating a tert-butyl group [57,58,59]. All have the same molecular geometric skeleton, and, structurally, they only differ in the number and position of the hydroxyl groups [13,60] (Figure 2). In their pure form, ginkgolides consist of odorless white crystals or powders, with a bitter taste. They are relatively stable substances, which have a large number of functional groups and, therefore, have high melting points, with significant differences in the described values, due to the solvents used in crystallization, purity, and experimental conditions. These diterpenes are soluble in acetone, ethanol, methanol, ethyl acetate, tetrahydrofuran, dioxane, acetic acid, trifluoroacetic acid, acetonitrile, pyridine, and dimethyl sulfoxide, being sparingly soluble in ether and water. They are insoluble in hexane, benzene, chloroform, and carbon tetrachloride [58]. Ginkgolide B (molecular weight = 424.40 g/mol; octanol/water partition coefficient, log P = 1.72) is the most potent of all these diterpenes, being considered the most important for the biological activity of this plant [58].

**Figure 3:**
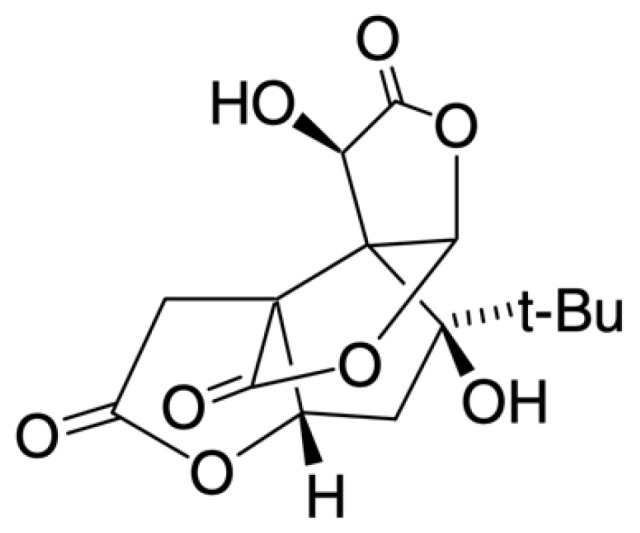
Chemical structure of bilobalide.

Bilobalide (molecular weight: 326.30 g/mol) (Figure 3) corresponds to a sesquiterpene, and is considered the main terpenoid found in Ginkgo biloba leaves [57,58,61]. Like ginkgolides, bilobalide also has a bitter taste, as well as a high melting point (~300 °C). Although specific data on the solubility of bilobalide are not available, its polarity and solubility appear to be similar to those exhibited by ginkgolides, with the exception of being unstable for pH values above 7. Due to their high solubility in acetone-water and methanol-water mixtures, ginkgolides and bilobalide are often extracted from Ginkgo biloba leaves using these mixtures [58]. Both ginkgolides and bilobalide are the only natural compounds in which a tert-butyl group is present [61].

After oral administration, flavonol glycosides are rapidly hydrolyzed by the intestinal microflora into aglycones, metabolized (mainly glucuronidation; methylation, sulfation, and oxidative degradation may also occur), and their pharmacologically active metabolites are absorbed [61]. In contrast, ginkgolides and bilobalide are absorbed as parent compounds, being readily detectable in human plasma soon after their oral administration [42,61]. After oral administration, ginkgolides A and B, as well as bilobalide, exhibit higher levels of systemic exposure than ginkgolides C and J. However, after intravenous administration, all of these terpene trilactones exhibit high levels of exposure. The significant differences between compounds in oral bioavailability can be attributed to differences in intestinal absorption between these terpenoids. The oral bioavailability of ginkgolides A and B is greater than that of bilobalide, with these compounds having greater oral bioavailability than ginkgolides C and J (6–15%) [62]. Terpene trilactones reach peak plasma concentrations very quickly. Ginkgolide B reaches this concentration a little later than either ginkgolide A or bilobalide, the latter having a significantly shorter half-life than ginkgolide B [42,61]. The half-lives of ginkgolides and bilobalide are found to be shorter when they are administered intravenously, compared to when they are administered orally. These compounds have been shown to easily reach extracellular and intracellular therapeutic targets. Renal excretion based on glomerular filtration is the main route of elimination for bilobalide and ginkgolides, whose unbound fractions, of the latter, in plasma account for 45–92% [62].

The effect of Ginkgo biloba on CYP450 isoforms is incompletely understood. EGb significantly inhibits CYP2C9, the isoform that metabolizes warfarin, in vitro but not in vivo [63]. Similarly, EGb does not affect CYP2D6 or CYP3A4 in vivo [64].

## 4. Cardiovascular Activity of Ginkgo Biloba and Its Main Compounds

### 4.1. Antioxidant Activity

Several studies have shown that EGb exhibits significant antioxidant activity in vitro in a wide range of tissues from animal models and human subjects. This antioxidant activity has been attributed to both terpenoids ginkgolides and bilobalide, as well as to flavonoids [65,66]. Overall, EGb is known to increase the levels of glutathione, to increase the activity of superoxide dismutase, and to decrease the expression and activity of NADPH oxidase, whereas decreasing the levels of malondialdehyde [67]. More recently it has also become apparent that the antioxidant activity of EGb can also be attributed to the activation of the Akt/Nrf2 signal pathway, which is in part responsible for the regulation of the cellular resistance to oxidative stress [68,69]. These activities lead to a decrease in the levels of reactive oxygen and nitrogen species, thus preventing lipid and protein peroxidation, respectively, thereby decreasing cellular damage. With regards to cardiovascular disease, EGb has been shown to reduce the oxidative stress of cardiomyocytes in animal models of myocardial injury [68,70,71], atherosclerosis [72], hypertension by kidney damage [73], as well as in animals and human subjects with metabolic syndrome [74,75,76].

Ginkgo biloba displays an important pro-endothelial activity both in vitro and in vivo, being largely attributed to its antioxidant activity, which prevents the destruction and inactivation of secreted nitric oxide (NO) [77]. In addition, it is known that Ginkgo biloba also induces the expression of endothelial NO synthase (eNOS) when incubated with human umbilical vein endothelial cells (HUVECs) in vitro, which consequently increases NO [78]. Furthermore, Ginkgo biloba also increases the number of endothelial progenitor cells in peripheral blood, as well as their migration ability, adhesiveness, and in vitro vasculogenesis activity [79]. This pro-endothelial activity also seems to result from a direct modulation of Ginkgo biloba on the calcium homeostasis of endothelial cells. One study showed that Ginkgo biloba could inhibit type 4 phosphodiesterase by decreasing agonist-induced calcium enhancement of calcium, again suggesting the importance of this ion in the normal function of endothelial cells [80]. Considering the increasingly recognized central role of the endothelium in cardiovascular physiology and pathophysiology [81], this pro-endothelial activity of Ginkgo biloba constitutes an important mechanism for decreasing cardiovascular risk.

### 4.2. Cardiac Activity In Vitro and Ex Vivo

Ginkgo biloba is known to modulate the activity of several ion channels on cardiomyocytes in vitro. For example, EGb decreases the maximum voltage of the ventricular action potential and shortens its duration [82]. These actions are attributed to the inhibition of calcium channels, delayed rectifier potassium channels, and of inward rectifier potassium channels [83]. In contrast, bilobalide also decreases maximum voltage, but shortens the action potential duration and enhances the calcium and delayed rectifier potassium currents. In addition, another study has demonstrated that EGb inhibits hyperpolarization-activated cyclic nucleotide-gated channels 2 and 4, being more potent in the latter, present in the sinoatrial node and ventricles [84]. These results suggest that multiple bioactive compounds of Ginkgo biloba are able to differently modulate cardiomyocyte ion channels, therefore exerting different effects on cardiac electrophysiological properties.

In a rat model of D-galactose-mediated ventricular ageing, treatment with EGb 761^®^ significantly reduced intracellular calcium concentration during diastole and increased its reuptake by means of sarcoplasmic endoplasmic receptor calcium ATPase, therefore improving diastolic dysfunction [85]. In an electrocardiography (ECG) study of guinea pig hearts, EGb and ginkgolide A increased the PR interval, indicating a calcium channel-blocking activity [86]. In contrast, the PR interval was reduced by ginkgolide B. In addition, both ginkgolides A and B and bilobalide reduced the QT interval, suggesting the activation of potassium channels, whereas EGb resulted in an increased QT interval. Both EGb and ginkgolide B reduced the heart rate. The atrioventricular block was observed with EGb, ginkgolide A, and bilobalide, which suggests an arrhythmogenic potential that should be better characterized.

### 4.3. Vasorelaxant Activity Ex Vivo

**Figure 4:**
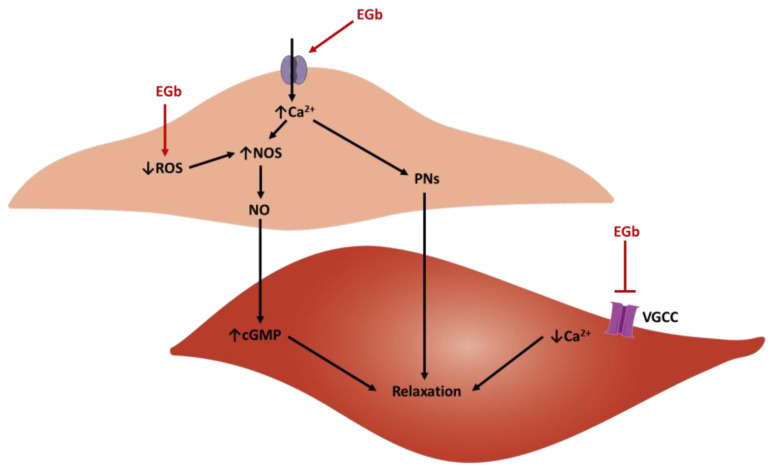
Scheme of the probable vasorelaxation mechanisms of the extracts of Ginkgo biloba (EGb). An endothelial cell is represented on top and a vascular smooth muscle cell at the bottom (cGMP—cyclic guanosine monophosphate; NO—nitric oxide; NOS—nitric oxide synthase; PNs—prostanoids; ROS—reactive oxygen species; VGCC—voltage-gated calcium channel).

Ginkgo biloba and some of its compounds have shown significant vasorelaxant activities in humans [87] and in different animal species, namely rats [88,89,90,91], pigs [92,93], and rabbits [94]. The main results of these studies are presented in Table 2 and the probable vasorelaxant mechanisms are represented in Figure 4.

EGb evoked a concentration-dependent relaxation of norepinephrine (NE)-precontracted aortae of Wistar rats [88,89,90]. This response was reported to have been significantly inhibited by L-nitro-methyl-arginine (NMMA, i.e., nitric oxide synthase inhibitor) but not by tetraethylammonium (TEA, i.e., voltage-gated potassium channel blocker), suggesting that NO is an important mediator, whereas the potassium efflux-mediated hyperpolarization of vascular smooth muscle (VSM) was not considered relevant [88]. Later, the same authors identified that EGb-mediated vasorelaxation is age-dependent, with a lower response intensity being observed in the aortae of older animals, although this finding was not statistically significant [90]. In addition to EGb, this vasorelaxation response was also identified for isolated ginkgolides A, B, and C as well as bilobalide, with an increasing potency in that order [89]. Results from Wistar-Kyoto rats (WKYRs) and spontaneously hypertensive rats (SHRs) seem to support this pharmacological activity. In animals that received oral supplementation for 30 days, EGb significantly enhanced the vasorelaxant activity of acetylcholine (ACh) in SHRs but not in WKYRs [95]. This effect was corroborated by the significant increase in ACh-mediated calcium uptake in SHR but not in WKY. In contrast, EGb did not affect the vasorelaxant activity of sodium nitroprusside (SNP, i.e., an NO donor) in any group [95]. This suggests that EGb vasorelaxation is strain-dependent, probably related to NO levels (lower in SHRs), and might be mediated by the promotion of both NO and prostanoids, even though the latter hypothesis was not tested. A subsequent study revealed that in aged SHRs, a 30-day supplementation did not induce vasorelaxation in phenylephrine (PE, i.e., alpha-1-adrenoreceptor blocker)-precontracted aortae or significantly improve the vasorelaxant activity of ACh, SNP or isoproterenol [96]. These results suggest that EGb loses efficacy with age irrespectively of the blood pressure profile of the animals. In Wistar rats subjected to the two-kidney-one-clip (2K,1C) model of hypertension, a 3-week EGb supplementation caused a significant vasorelaxation of NE-precontracted aortae and a significant potentiation of ACh-mediated vasorelaxation. However, it did not potentiate vasorelaxation by SNP, suggesting that it might evoke the release of endothelial prostanoids and/or interfere with the pathways involving calcium in VSM cells [97].

In the rat mesenteric arteries, a 10-day supplementation with EGb evoked a concentration-dependent relaxation of potassium chloride (KCl)- and PE-precontracted vessels, together with the significant potentiation of SNP-mediated relaxation [91]. These results suggest that EGb might block voltage-gated calcium channels or prevent the release of intracellular calcium stores, besides potentiating the release of NO by the endothelium. However, a 4-week dietary supplementation with EGb did not change the PE-mediated contraction or the ACh-mediated vasorelaxation in aged SHRs [96]. In the corpus cavernosum of New Zealand rabbits, EGb increased the vasorelaxant activity of mirodenafil (i.e., a phosphodiesterase-5 inhibitor) [94]. In addition, EGb-mediated vasorelaxation was inhibited by TEA, suggesting that in this tissue, potassium efflux-mediated VSM hyperpolarization is an important mechanism. In porcine basilar arteries, EGb evoked a concentration-dependent relaxation of intact and endothelium-denuded vessels, suggesting that endothelium is not essential for vasorelaxation. However, EGb did not affect SNP-mediated relaxation. In addition, EGb enhanced transmural nerve stimulation-mediated vasorelaxation, which was abolished by tetradotoxin (TTX, i.e., voltage-gated sodium channel blocker) and L-N-arginine (i.e., NO precursor). Taken together, these results show that in these vessels, EGb evokes vasorelaxation by directly acting on VSM as well as enhancing the release of nerve-derived NO [93]. Finally, in human umbilical arteries, EGB 761^®^ evoked relaxation of serotonin (5-HT) -precontracted vessels, a response that was inhibited by indomethacin (i.e., nonselective cyclooxygenase inhibitor) and N^ω^-nitro-L-arginine methyl ester (L-NAME, i.e., nitric oxide synthase inhibitor), suggesting that the endothelial secretion of both nitric oxide and of prostanoids is important for vasorelaxation [87].

### 4.4. Vasoconstrictor Activity Ex Vivo

In a minor subset of studies, EGb has been shown to evoke vasoconstriction ex vivo. EGb potentiated the NE-induced aortic contraction of New Zealand rabbits [99], suggesting a potential stimulatory action on the release of catecholamines or an inhibitory activity over monoamine oxidase (MAO) enzymes [100]. Effectively, Ginkgo biloba has been shown to inhibit MAO enzymes in rats due to the presence of kaempferol [101]. A subsequent study showed similar vasoconstrictor activity in the rabbit vena cava, which was partly blocked by phenoxybenzamine (i.e., alpha-1-adrenoreceptor blocker), suggesting an antagonistic action on alpha-1 receptors [102]. Taken together, ex vivo studies revealed that the vasoactive effects of EGb are species- and strain-dependent.

### 4.5. Anti-Hypertensive Activity of EGb In Vivo—Animal Models

Several studies have been published so far on the effects of Ginkgo biloba on the blood pressure profile of normotensive animals [98], as well as in different hypertensive animal species and strains, such as L-NAME-mediated hypertension [73,103], 2K,1C rats [97], deoxycorticosterone acetate (DOCA)-salt hypertensive rats [104], SHRs [95], and SHRSP/Izm rats [105]. The main results of these studies are presented in Table 3.

Published data suggest that this activity is age- and strain-dependent. In normotensive Sprague-Dawley rats, the intravenous administration of EGb reduced blood pressure over a period of 5 min, and was attenuated by pretreatment with L-NAME, again suggesting the prominent role of NO in this cardiovascular response [98]. Similar results from the oral administration of EGb in WKYRs have been reported. One study reported that a 20-day administration failed to change blood pressure, although it lowered heart rate [104]. Another reported that the 30-day oral administration of EGb failed to change blood pressure in young normotensive WKYRs [95]. This suggests that the cardiovascular response to Ginkgo biloba is practically irrelevant in normotensive animals, confirming ex vivo studies, probably due to the fact that these animals do not demonstrate a decrease in NO release.

In contrast, Ginkgo biloba has demonstrated anti-hypertensive efficacy in several hypertensive animal models. A 30-day oral administration of EGb lowered systolic blood pressure in young SHRs [95]. In L-NAME-mediated hypertensive Wistar rats, the administration of EGb from the ninth week of hypertension induction caused a significant decrease in systolic, diastolic, and mean blood pressure relative to controls [103]. Similar results were noted in L-NAME-mediated hypertensive Wistar rats with hypercholesterolemia [73]. In both studies, the authors proposed that EGb lowered blood pressure not only through vasodilation but also by an antioxidant-mediated improvement in renal function. In 2K,1C hypertensive rats, the administration of EGb for 3 weeks produced a dose-dependent decrease in blood pressure with no significant change in heart rate [97]. These effects were attributed to different activities, including inhibition of renin-angiotensin-aldosterone axis, endothelial restoration, and vasorelaxation. In DOCA-salt hypertensive rats a 20-day administration of 2% EGb decreased blood pressure and heart rate. The anti-hypertensive activity of EGb was more pronounced during the daytime, a resting period for rats, however, the bradycardic effect was time-independent [104]. Similarly, in stroke-prone SHRSP/Izm animals, a 3-week oral administration (60 and 120 mg/kg) significantly decreased blood pressure [105]. This administration increased the urinary excretion of NO metabolites, nitrite/nitrate, and urinary 8-hydroxy-2′-deoxyguanosine, as well as increasing the mRNA expression of eNOS. These results again support the endothelium-dependent vasodilation activity of EGb. In contrast, in aged SHRs, the oral administration decreased heart rate and blood flow velocity although a significant level was only observed after 30 days [96]. Blood pressure and the endothelial content of NOS and guanylyl cyclase, however, remained unchanged throughout the experimental period.

### 4.6. Effects of Ginkgo biloba on Blood Pressure and Heart Rate in Healthy Subjects—In Vivo Studies

Several studies have recorded data on the short-term effects of Ginkgo biloba on the blood pressure of healthy subjects, with the main results being resumed in Table 4. For example, a group of young healthy subjects enrolled in a study to assess the effects of Ginkgo biloba on ocular vessels. The results showed no change in blood pressure 3 h after taking 240 mg EGb 761^®^ extract when compared to a placebo [106]. In a different study the oral administration of 360 mg EGb 761^®^ did not change heart rate, blood pressure, or arterial pulse characteristics over a period of 6 h when compared with a placebo. The only exception noted was a significant increase in the stiffness index 2 h post-administration [107]. Thirdly, a group of healthy subjects took 120 mg EGb for 2 days. No significant changes in blood pressure or heart rate were noted against the placebo [108]. In a fourth study, a group of young healthy subjects received 120 mg EGb daily for 5 days. On day 5 of the experiment, both heart rate and systolic blood pressure were lower than on previous days [109]. In another group of young healthy subjects, 240 mg of EGb was given for 7 days, and no change in blood pressure, heart rate, or electrocardiographic parameters were noted when compared with the placebo [110]. Finally, a group of healthy subjects was examined before and after a 3 month treatment course with Ginkgo biloba 120 mg/day, and a significant decrease in systolic and diastolic blood pressure was reported [109].

These results suggest that the hemodynamic effects of Ginkgo biloba are both dose- and time-dependent. However, the variability between these studies can also be attributed to differences in their design, namely imbalances in terms of the male-to-female ratio and the probable variability in terms of the menstrual cycle, a known variable that influences hemodynamics [111]. These studies were carried out in the absence of challenge tests. In a different study, a group of young healthy subjects received EGb 761^®^ or the placebo orally. Both groups were subjected to stress tests (mental stress and handgrip static exercise) before and after taking the extract [112]. Results showed that Ginkgo biloba significantly prevented the rise in blood pressure evoked by stress tests when compared to a placebo. In addition, the extract also prevented the increase in blood cortisol in males. Although these results are interesting and suggest that Ginkgo biloba may also suppress the hypothalamus-hypophysis-adrenal axis, they may also lack reproducibility. According to the authors, this study was carried out during a regular university course and not in an experimental environment, which may affect the results. Finally, in a group of 60 elderly subjects with mild-to-moderate cognitive impairment, a treatment course of Ginkgo biloba 120 or 240 mg daily for 3 months, resulted in a significant decrease in diastolic blood pressure in the low-dose group [113].

### 4.7. Effects of Ginkgo biloba on Organ Perfusion in Healthy Subjects—In Vivo Studies

#### 4.7.1. Effect on Skin Perfusion

The effects of Ginkgo biloba extracts on cutaneous perfusion have been explored in healthy subjects in only a few studies (Table 5). When a Ginkgo biloba extract (Gibidyl forte^®^), containing 9.6 mg flavone glucoside and 2.4 mg terpenlactones) was administered three times daily to a group of young healthy subjects for 6 weeks, it significantly increased forearm perfusion on weeks 3 and 6 without changing blood pressure [115]. In contrast, a 3-week treatment course with 240 mg/day (80 mg three times daily) in a group of healthy middle-aged subjects on the forefoot resulted in a significant decrease in mean skin perfusion, both basal and post-occlusion, when compared to placebo [116]. These opposing results can be attributed to differences in quantification techniques, treatment course, dose, and, eventually, body posture during measurements. Nonetheless, the study carried out in middle-aged subjects was able to better characterize the vascular effects. Despite the mean response having been a perfusion decrease, it was also heterogeneous, having been observed in seventeen subjects, whereas three showed no change and seven a slight increase. Such a thorough evaluation was not achieved in the study of the young subjects. Furthermore, the subjects with higher baseline perfusion showed the highest decrease and vice-versa. Interestingly, these skin perfusion results were strongly correlated with the urinary Ginkgo biloba metabolites, with the metabolic fingerprinting depending on the direction of the perfusion change rather than being related to the intervention. These results suggest that the vascular response to Ginkgo biloba may be related to individual metabolism.

#### 4.7.2. Effect on Coronary Perfusion

To the author’s knowledge, only one study investigated the effects of Ginkgo biloba on coronary perfusion in humans (Table 5). In a group of middle-aged healthy subjects who received an intravenous administration of Ginkgo biloba, perfusion in the left anterior descending coronary artery, assessed with transthoracic Doppler echocardiography, increased significantly [117]. Since this administration also significantly increased the flow-mediated dilation of the brachial artery, the vasodilation of the coronary artery was attributed to a potentiation of the endothelial function.

#### 4.7.3. Effect on Cerebral Perfusion

Several studies on Ginkgo biloba and cerebral perfusion have been carried out so far (Table 5). In a small group of healthy middle-aged subjects, a 4-week treatment course with 60 mg twice-daily significantly increased cerebral perfusion assessed by dynamic susceptibility contrast-enhanced magnetic resonance imaging. Although a small but significant increase in perfusion was observed globally, both in grey and white matter, a more pronounced increase was noted on the left parietal–occipital white matter [118].

#### 4.7.4. Effect on Ocular Perfusion

Different studies have explored the effects of EGb on ocular hemodynamics in both healthy patients and patients with glaucoma but with no cardiovascular disease (Table 5). One study showed that a 240 mg single administration significantly increased the end-diastolic flow velocity of the ophthalmic artery [108]. In contrast, a more recent study showed that a similar dose failed to evoke significant vascular responses when compared with a placebo [106]. In glaucoma patients, EGb also increases ocular blood flow. In patients with open-angle glaucoma, a one-month treatment course with a daily antioxidant supplement containing 120 mg EGb (among other compounds) resulted in the increase of the peak systolic and/or diastolic blood velocities of different ocular vessels (retrobulbar, superior and inferior temporal retinal capillary vessels) when compared with the placebo [121]. Similarly, in patients with normal angle glaucoma and a one-month treatment course with 80 mg EGb twice daily resulted in an increase in peripapillary perfusion [122].

#### 4.7.5. Effect on Cochlear Perfusion

Ginkgo biloba is known to improve cochlear microcirculation in animals, with and without vascular dysfunction (Table 5). Administration of EGb 761^®^ to adult guinea-pigs for 6 weeks showed no significant difference in the vascular density on the stria vascularis and spiral lamina on post-mortem analysis when compared with controls. Treatment for 4 or 6 weeks also attenuated the decrease in blood pressure brought by sodium salicylate and improved vascular conductance. Furthermore, EGb partly counteracted the salicylate-mediated reduction in cochlear perfusion and increased hypoxia-mediated perfusion increase [119]. Due to this effect of cochlear vasodilation, Ginkgo biloba is able to increase the cochlear uptake of ototoxic drugs such as aminoglycosides. However, studies are not consistent on the effect of Ginkgo biloba in aminoglycoside-mediated cochlear injury since this increased uptake and ototoxicity might be opposed by the antioxidant effects [123,124,125]. More recently, in a guinea pig model of lipopolysaccharide-mediated otitis media and labyrinthitis, administration of Ginkgo biloba prevented the decrease in cochlear perfusion and hair cell damage [120].

These beneficial effects on cochlear perfusion seem to justify the exploration of Ginkgo biloba on sensorineural hearing loss and tinnitus. The majority of clinical studies published so far have shown no significant efficacy of Ginkgo biloba when compared with a placebo for the improvement of tinnitus and/or hearing loss [19,20]. However, in animal studies aimed at preventing or treating cochlear damage (drug- or noise-induced), Ginkgo biloba showed encouraging results. For example, when animals received Ginkgo biloba prior to noise exposure, their cochlear synaptopathy was not as pronounced as in animals that had not received this drug [126]. Even though these effects have not been consistently demonstrated in clinical studies, results from animal models have shown that Ginkgo biloba also attenuated hearing loss when administered after noise-induced trauma [127,128]. These beneficial effects are probably attributed to vasodilatory and antioxidant activities of EGb which prevent the energy depletion and oxidative stress of hair cells which is a hallmark of the pathophysiology of noise-induced hearing loss [129,130]. This hypothesis is supported by previous studies that have already demonstrated that antioxidant-rich diets or antioxidant supplementation have preventive effects on the morphological and functional changes to the inner ear caused by age- and noise-induced damage, in some cases slowing the progression of hearing loss [131,132].

## 5. Adverse Reactions from Ginkgo biloba

Several adverse effects of Ginkgo biloba have been reported, although of not great statistical significance. In one patient, Ginkgo biloba caused palpitations with accompanying ventricular arrhythmia, namely an ECG pattern compatible with left bundle branch block premature ventricular depolarization pattern, which resolved with the discontinuation of the drug [133]. In another paper, a 35-year-old woman developed paroxysmal atrial fibrillation after a 2-month treatment with EGb 240 mg/day [134]. One patient who was elderly, hypertensive, and under thiazide medication developed severely high blood pressure after starting Ginkgo biloba, which subsided after discontinuation [135]. In addition, two patients with well-controlled epilepsy presented with recurrent seizures within 2 weeks of commencing EGb, which ceased after discontinuation [136].

Considering that ginkgolides, particularly ginkgolide B, are able to inhibit platelet-activating factors [137], it is not surprising that Ginkgo biloba has been implicated in spontaneous intracerebral and subdural hemorrhages. A healthy 33-year-old woman with a 2- year long history of supplementing with 120 mg Ginkgo biloba daily developed a bilateral subdural hemorrhage [138]. Secondly, an elderly female patient with a history of multiple cardiovascular issues treated with warfarin developed intracerebral parietal hemorrhage after a 2-month treatment with Ginkgo biloba [139]. Similarly, another paper reported the case of a 72-year-old woman who was taking 100 mg Ginkgo biloba daily for 6–7 months and presented with a left frontal subdural hematoma [140]. Finally, a 61-year-old male patient, previously healthy, developed a subarachnoid hemorrhage after more than 6 months of taking 40 mg tablets, three to four times daily [141]. Such rare associations between EGb and warfarin are in line with the prevalence of mutant CYP2C9 alleles, although a potential effect from impurities in certain commercial brands cannot be excluded [63]. These effects are expected considering the different molecular targets of Ginkgo biloba, however, they are not statistically significant. In addition, it is advised that Ginkgo biloba should be used with caution during pregnancy [142].

## 6. Conclusions

Ginkgo biloba is a plant with a long history of medical use for diverse health problems. For cardiovascular diseases, systematic reviews and meta-analyses of clinical studies have shown that this plant is useful in only a few conditions. Still, extracts of Ginkgo biloba are among the most sold supplements worldwide, which justifies the need for a deeper knowledge of its cardiovascular effects, especially in healthy subjects, whose data has been considerably undervalued. Ginkgo biloba displays a modulatory effect on cardiac function by acting in different ion channels on cardiomyocytes. It also displays vasorelaxant activity, attributed to the potentiation of the endothelial release of nitric oxide and prostanoids, in addition to the blockage of calcium channels on vascular smooth muscle. In healthy humans, Ginkgo biloba increases perfusion in different vascular beds, namely the ocular, cochlear, cutaneous, cerebral, and coronary, without significantly affecting blood pressure. Although scarce, recent evidence suggests that individual metabolism of Ginkgo biloba in different subjects is an important determinant of the nature and magnitude of vascular response. Collectively, Ginkgo biloba is considered to be generally safe, with a low frequency of adverse reactions. In the future, better controlled clinical studies should be performed in order to identify the target populations who may benefit the most from pharmacotherapeutic interventions involving Ginkgo biloba.

## Figures and Tables

**Table 1:** Main classes of chemical compounds present in EGb 761^®^ (Adapted from [47]).

Compound Class	Percentage (%)
Flavonol glycosides	24
Non-flavonol glycosides	20
Proanthocyanidins	7
Terpenes	6
Catechins	2
Carboxylic acids	13
Other compounds (organic, inorganic, unknown)	28

**Table 2:** Results of the vasorelaxant activity of Ginkgo biloba ex vivo (5-HT—serotonin; DA—dopamine; EGb—extract of Ginkgo biloba; GKA—Ginkgolide A; L-NAME—N^ω^-nitro-L-arginine methyl ester; KCl—potassium chloride; L-NMMA—N^G^-methyl-L-arginine; m.o.—months old NE—norepinephrine; PE—phenylephrine; SHRs—spontaneously hypertensive rats; SNP—sodium nitroprusside; TEA—tetraethylammonium; TTX—tetrodotoxin; WKYRs—Wistar-Kyoto rats; w.o.—weeks old).

Authors	Species and Strain	Type of Vessel	Compound and Dose/Concentration	Main Results
Nishida and Satoh (2003) [88]	Male Wistar rats (4–10 w.o.; undisclosed weight)	Aorta	EGb (0.3–3 mg/mL) and bilobalide (0.1–100 μmol/L)	EGb and bilobalide relaxed NE-precontracted intact vessels. EGb-mediated relaxation was significantly inhibited by L-NMMA but not by indomethacin or TEA. Bilobalide-mediated relaxation was inhibited in a calcium-free medium or by pretreatment with L-NMMA.
Nishida and Satoh (2004) [89]	Male Wistar rats (4–15 w.o.; undisclosed weight)	Aorta	EGb (Ginkgolon-24^®^, 0.03–3 mg/mL) and its isolated terpenoids and flavonoids (0.1–100 μmol/L)	EGb, terpenoids (ginkgolides A-C, bilobalide), and flavonoids (quercetin, rutin) relaxed NE-precontracted intact vessels.
Nishida and Satoh (2005) [90]	Male Wistar rats (5 to 25 w.o.; undisclosed weight)	Aorta	EGb (0.1–3 mg/mL and bilobalide (0.1–100 μmol/L)	Concentration-dependent relaxation of NE-precontracted intact vessels, with response intensity decreasing with the animals’ age.
Kubota et al. (2006) [95]	Male WKYRs and SHRs (6 w.o.; undisclosed weight)	Aorta	EGb 0.05–0.5% orally for 30 days	No vascular response in vessels from WKYRs. Dose-dependent potentiation of acetylcholine-mediated vasorelaxation in vessels from SHRs.
Koltermann et al. (2007) [98]	Male Sprague-Dawley rats (180–220 g; undisclosed age)	Aorta	EGb 761^®^ 5 mg i.v.	Relaxation of precontracted vessels.
Auguet et al. (1982) [99]	Male New Zealand rabbits (1.8–2.6 kg; undisclosed age)	Aorta	EGb 100 μg/mL	Potentiation of NE-induced contraction; no effect on 5-HT- or desipramine-induced contraction.
Laukeviciene et al. (2012) [91]	Wistar rats (undisclosed sex, age and weight)	Mesenteric artery	EGb 0.32 mL/kg/day orally for 10 days	Relaxation of KCl- and PE-precontracted vessels; potentiation of SNP-mediated relaxation.
Kubota et al. (2007) [96]	Male SHRs (50 w.o.)	Mesenteric artery	EGb 4-week supplementation	No change in PE-precontracted vessels or in ACh-mediated relaxation.
Zhou et al. (2006) [92]	Pigs (6–7 m.o.)	Coronary artery	GKA	GKA recovered bradykinin-mediated relaxation in arteries incubated with homocysteine. However, GKA did not modify maximum contraction or relaxation evoked by U46619 or SNP.
Chen et al. (1997) [93]	Pigs (undisclosed age and weight)	Basilar artery	EGb from leaves (15–90 μg/mL) and gingenosides fraction (20–120 μg/mL)	Concentration-dependent relaxation of intact and endothelium-denuded arteries, with and without TNS-induced relaxation. The latter was inhibited by N-L-arginine and by TTX but not by SNP.
Kim et al. (2011) [94]	New Zealand rabbits (22–26 w.o., 3–4 kg)	Corpus cavernosum	EGb	Relaxation of NE-precontracted tissue by EGb or mirodenafil. This response was inhibited by TEA.
Gokbas (2021) [87]	Human subjects (healthy full-term deliveries)	Umbilical artery	EGb 761^®^ (50–500 μg)	Relaxation of 5-HT-precontracted intact vessels. This response was inhibited by indomethacin and L-NAME.

**Table 3:** Results of the anti-hypertensive activity of Ginkgo biloba in vivo (DOCA—deoxycorticosterone acetate; EGb—extract of Ginkgo biloba; i.v.—intravenous; L-NAME—N^ω^-nitro-L-arginine methyl ester; SHRs—spontaneously hypertensive rats; SHRSP/Izm—SHRs—spontaneously hypertensive stroke-prone rats; WKYRs—Wistar-Kyoto rats).

Authors	Species and Strain	Compound and Dose/Concentration	Main Results
Koltermann et al. (2007) [98]	Male Sprague-Dawley rats (180–220 g, undisclosed age)	EGb 761^®^ 5 mg i.v.	Significant decrease of systolic blood pressure, inhibited by L-NAME.
Abd-Eldayem et al. (2016) [103]	L-NAME-induced hypertensive Wistar rats (undisclosed age and weight)	EGb 761^®^ 100 mg/kg/day orally for 3 weeks	Significant decrease of blood pressure.
Abdel-Zaher et al. (2017) [73]	L-NAME-induced hypertensive and hypercholesterolemic Wistar rats (undisclosed age and weight)	EGb 761^®^ 100 mg/kg/day for 3 weeks	Significant decrease of blood pressure.
Umegaki et al. (2000) [104]	DOCA-salt hypertensive rats (undisclosed age and weight)	EGb 2% orally for 20 days	Significant decrease of blood pressure and heart rate.
Kubota et al. (2007) [96]	Male SHRs (N = 6, 50 w.o., undisclosed weight)	0.5% orally for 4 weeks	No change in systolic or diastolic blood pressure; significant reduction in heart rate and blood flow velocity.
Sasaki et al. (2002) [105]	SHRSP/Izm rats (6 w.o., undisclosed weight)	EGb 761^®^ (60 and 120 mg/kg) orally for 3 weeks	Significant decrease in blood pressure.
Mansour et al. (2011) [97]	Male Wistar albino rats (120–140 g, undisclosed age)	EGb 761^®^ (180 mg/kg/day) orally for 3 weeks)	Significant decrease in blood pressure; no change in heart rate.

**Table 4:** Main results of the clinical studies on the effects of Ginkgo biloba on the blood pressure of healthy human subjects (EGb—extract of Ginkgo biloba; ECG—electrocardiography; y.o.—years old).

Authors	Study Sample	EGb Composition	Concentration/Dosage and Duration of Treatment	Main Results
Chung et al. (1999) [108]	Healthy (N = 11; 8 females, 3 males; 10–61 y.o., mean 34 ± 3 y.o.)	EGb 761^®^	120 mg/day orally for 2 days	No change in heart rate and blood pressure when compared with the placebo.
Kudolo et al. (2000) [107]	Healthy (N = 20, 14 females, 6 males, 21–57 y.o.)	EGb 761^®^	120 mg/day orally for 3 months	Significant decrease in systolic and diastolic blood pressure.
Keheyan et al. (2011) [114]	Healthy (N = 14, males)	EGb 761^®^	360 mg orally, single dose	No change in heart rate or blood pressure over a 6 h period when compared with placebo.
Moulton et al. (2001) [109]	Healthy (N = 30 males, mean age 20.57 y.o.)	Undisclosed	120 mg/day orally for 5 days	Significant decrease of heart rate and blood pressure on day 5 when compared to the previous days.
Kalus et al. (2003) [110]	Healthy (N = 15; 7 females, 8 males; 20–24 y.o., mean 22.9 ± 1.1 y.o.)	EGb 761^®^	240 mg (120 mg twice-daily) orally for 7 days	No change in heart rate, blood pressure, or ECG parameters when compared with the placebo.
Jezova et al. (2002) [112]	Healthy (N = 70; 37 females, 33 males; 20–30 y.o.)	EGb 761^®^ (40 mg/mL)	120 mg orally, single administration	Attenuation of stress-mediated (handgrip and mental stimuli) increase in blood pressure.
Winther et al. (1998) [113]	Mild to moderate cognitive cognitive impaired (N = 40, 20 per group; both sexes; 61–88 y.o.; undisclosed cardiovascular status)	Undisclosed composition	120 (20 subjects) or 240 mg/day (20 subjects) orally for 3 months	Significant decrease of diastolic blood pressure in the subjects treated with 120 mg/day.
Wimpissinger et al. (2007) [106]	Healthy male subjects (N = 15; mean 25 ± 3 y.o.)	EGb 761^®^	240 mg orally, single administration (57.6 mg ginkgoflavonglycosidesand 14.4 mg terpenlactones)	No change in blood pressure 3 h after administration when compared with the placebo.

**Table 5:** Main results of the clinical studies on the effects of Ginkgo biloba extract on organ perfusion in healthy human subjects and in animals (i.p.—intraperitoneal; i.v.—intravenous; LPS—lipopolysaccharide;y.o.—years old).

Authors	Study Sample	Compound Concentration/Dosage and Duration of Treatment	Main Results
Mehlsen et al. (2002) [115]	Healthy subjects (N = 16; both sexes; median 32 y.o.)	1 tablet of Gibidyl forte^®^ thrice daily per os for 6 weeks	Significant increase in forearm perfusion on weeks 3 and 6 without changing blood pressure.
Boelsma et al. (2004) [116]	Healthy middle-aged subjects	240 mg EGb 761^®^/day (80 mg thrice daily) orally for 3 weeks	Significant decrease in mean forefoot perfusion, both basal and post-occlusion, when compared to placebo.
Wu et al. (2008) [117]	Healthy middle-aged male subjects (N = 30; 54 ± 10 y.o.)	EGb 0.7 mg/min i.v. for 120 min	Significant increase in the perfusion of the left anterior descending coronary artery.
Mashayekh et al. (2011) [118]	Healthy middle-aged subjects	EGb 120 mg/day orally (60 mg, twice-daily) for 4 weeks	Significant increase in cerebral perfusion.
Chung et al. (1999) [108]	Healthy subjects (N = 11; both sexes, mean 34 ± 3 y.o.)	EGb 40 mg, thrice daily, orally for 2 days	Significant increase in the end-diastolic flow velocity of the ophthalmic artery.
Wimpissinger et al. (2007) [106]	Healthy male subjects (N = 15; mean 25 ± 3 y.o.)	EGb 761^®^ 240 mg orally	No signficant vascular change in comparison with the placebo.
Didier et al. (1996) [119]	Adult guinea pigs (N = 4, undisclosed age)	100 mg/kg/day EGb 761^®^ orally for 6 weeks	Attenuation of salicylate-induced decrease in cochlear perfusion; potentiation of hypoxia-mediated cochlear perfusion.
Jang et al. (2011) [120]	Adult male guinea pigs (N = 10; 250–300 g)	Ear instillation of 10 mg/kg EGb and of LPS, followed by i.p. administration of EGb 100 mg/kg/day for 3 days	Significant attenuation of LPS-mediated decrease in cochlear perfusion and hair cell damage.

## Data Availability

Not applicable.
